# Supplementary material for: HIV restriction factor APOBEC3G binds in multiple steps and conformations to search and deaminate single-stranded DNA
Source: eLife. 2019 Dec 18;8:e52649. doi: 10.7554/eLife.52649 (PMC6946564; doi:10.7554/eLife.52649)
Supplement: Figure 2—source data 1. [file elife-52649-fig2-data1.pdf]

k_{on}

A3G	Force (pN)	Average (1/s)	Standard Error	N
WT	10	0.0065	0.0014	5
WT	20	0.0085	0.0016	5
WT	35	0.0051	0.0010	5
WT	50	0.0032	0.0005	5
WT	65	0.0021	0.0008	5
WT	80	0.0021	0.0007	5

k_{off}

A3G	Force	Average (1/s)	Standard Error	N
WT	50	0.014	0.002	5
WT	65	0.012	0.003	3
WT	80	0.014	0.002	3
FW	20	0.013	0.002	5
FW	35	0.016	0.003	3
FW	50	0.015	0.002	4
FW	65	0.015	0.003	3
FW	80	0.014	0.004	3

Average rates, associated standard errors, and biological replications (N) for measurements of A3G binding to (kon) and dissociation from (koff) ssDNA as plotted in figure 2C&F.
