# Supplementary material for: HIV restriction factor APOBEC3G binds in multiple steps and conformations to search and deaminate single-stranded DNA
Source: eLife. 2019 Dec 18;8:e52649. doi: 10.7554/eLife.52649 (PMC6946564; doi:10.7554/eLife.52649)
Supplement: Figure 2—figure supplement 1—source data 1. [file elife-52649-fig2-figsupp1-data1.pdf]

k_{on}

A3G	Salt Concentration	Force (pN)	Average (1/s)	Standard Error	N
WT	50 mM Na	20	0.0085	0.0016	5
WT	50 mM Na + 10 mM Mg	20	0.0027	0.0008	5
WT	150 mM Na + 1 mM Mg	20	0.0015	0.0002	7

k_{off}

A3G	Salt Concentration	Force (pN)	Average (1/s)	Standard Error	N
FW	50 mM Na	20	0.013	0.002	5
FW	50 mM Na + 10 mM Mg	20	0.017	0.004	6
FW	150 mM Na + 1 mM Mg	20	0.016	0.001	5

Average rates, associated standard errors, and biological replications (N) for measurements of A3G binding to (kon) and dissociation from (koff) ssDNA in different buffer conditions as plotted in figure 2 supplement B&C.
