# Supplementary material for: HIV restriction factor APOBEC3G binds in multiple steps and conformations to search and deaminate single-stranded DNA
Source: eLife. 2019 Dec 18;8:e52649. doi: 10.7554/eLife.52649 (PMC6946564; doi:10.7554/eLife.52649)
Supplement: Figure 3—source data 1. [file elife-52649-fig3-data1.pdf]

Extension Change

Force	Average (nm/nt)	Standard Error	N
10	0.0172	0.0030	5
20	0.0168	0.0018	5
35	0.0103	0.0014	5
50	0.0052	0.0011	5
65	0.0046	0.0005	5
80	0.0045	0.0005	5

Average extension change, associated standard errors, and biological replications (N) for measurements of A3G binding to (kon) and dissociation from (koff) ssDNA as plotted in figure 3A.
