# Supplementary material for: HIV restriction factor APOBEC3G binds in multiple steps and conformations to search and deaminate single-stranded DNA
Source: eLife. 2019 Dec 18;8:e52649. doi: 10.7554/eLife.52649 (PMC6946564; doi:10.7554/eLife.52649)
Supplement: Figure 4—source data 1. [file elife-52649-fig4-data1.pdf]

Observed re-equilibration rate

Force	Average (1/s)	Standard Error	N
10	0.49	0.05	9
15	0.25	0.06	6
20	0.20	0.03	7
35	0.19	0.02	7
50	0.26	0.02	6
65	0.33	0.03	7
80	0.48	0.08	12

Average rates, associated standard errors, and biological replications (N) for measurements of re-equilibration of an A3G saturated ssDNA after force jump as plotted in figure 4C.
