# Supplementary material for: HIV restriction factor APOBEC3G binds in multiple steps and conformations to search and deaminate single-stranded DNA
Source: eLife. 2019 Dec 18;8:e52649. doi: 10.7554/eLife.52649 (PMC6946564; doi:10.7554/eLife.52649)
Supplement: Figure 5—source data 1. [file elife-52649-fig5-data1.pdf]

A3G binding to ssDNA-dsDNA hybrid construct at low forces

Force (pN)	Average ΔExtension (nm/nt)	Standard Error	Average Rate (1/s)	Standard Error	N
4	0.097	0.009	0.025	0.011	5
8	0.053	0.005	0.034	0.012	5
12	-0.032	0.007	0.098	0.035	5
16	-0.025	0.005	0.144	0.048	5

Average extension changes, rates, associated standard errors, and biological replications (N) for measurements of A3G binding to ssDNA at low forces as plotted in figure 5C&D.
