# Supplementary figures and images for: HIV restriction factor APOBEC3G binds in multiple steps and conformations to search and deaminate single-stranded DNA
Source: eLife. 2019 Dec 18;8:e52649. doi: 10.7554/eLife.52649 (PMC6946564; doi:10.7554/eLife.52649)

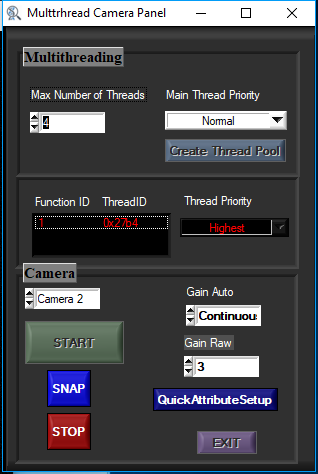

Supplement: Source code 1. [file elife-52649-code1.zip › UTC-master/CameraPanel.png]

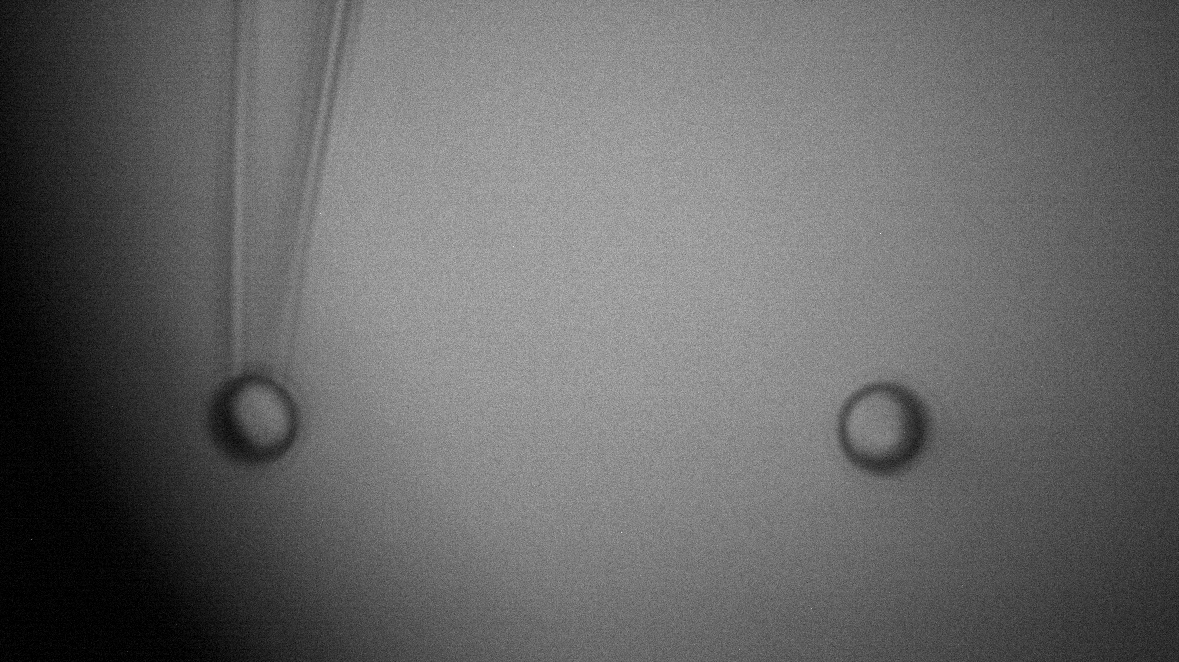

Supplement: Source code 1. [file elife-52649-code1.zip › UTC-master/CameraView.png]

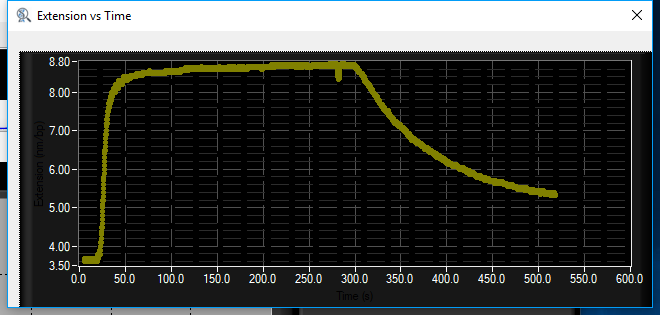

Supplement: Source code 1. [file elife-52649-code1.zip › UTC-master/Extension_timePanel.png]

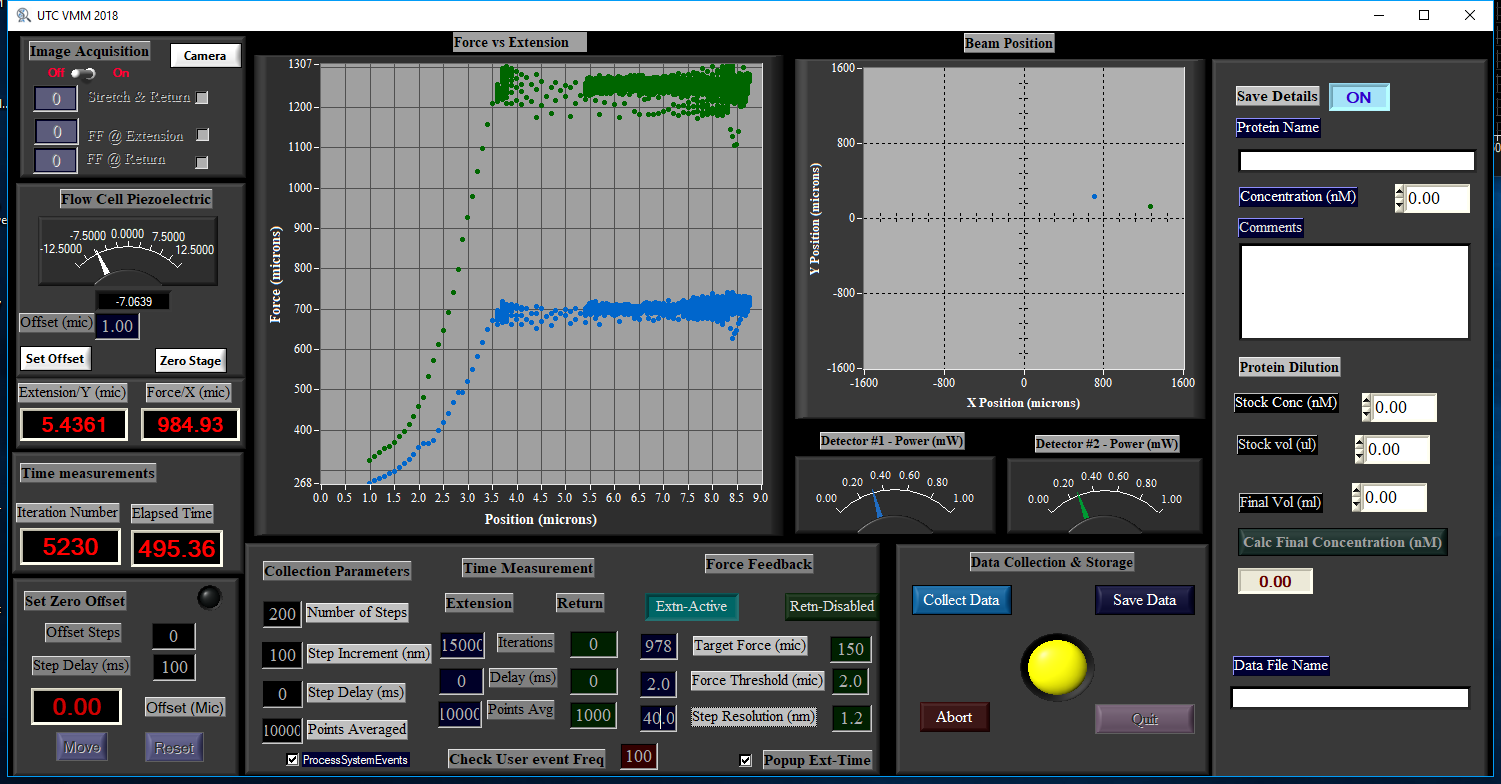

Supplement: Source code 1. [file elife-52649-code1.zip › UTC-master/MainPanel.png]

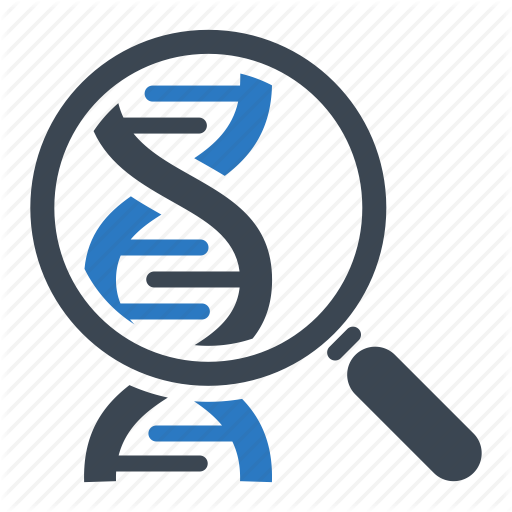

Supplement: Source code 1. [file elife-52649-code1.zip › UTC-master/UTC2017.png]

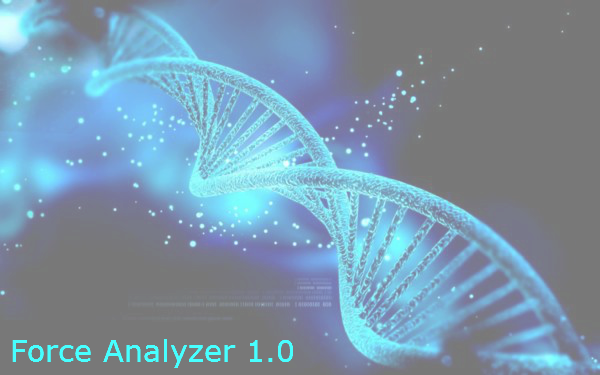

Supplement: Source code 2. [file elife-52649-code2.zip › Fexta2016-master/DNA splash.png]

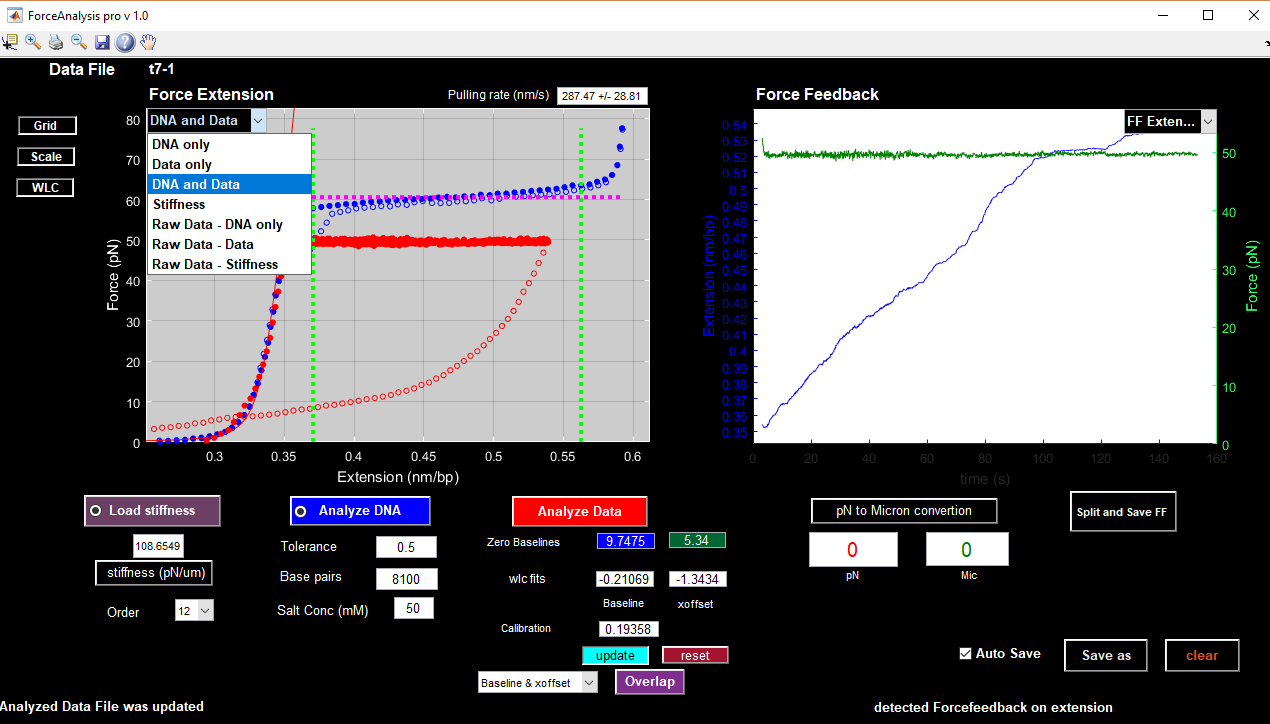

Supplement: Source code 2. [file elife-52649-code2.zip › Fexta2016-master/Gui.png]

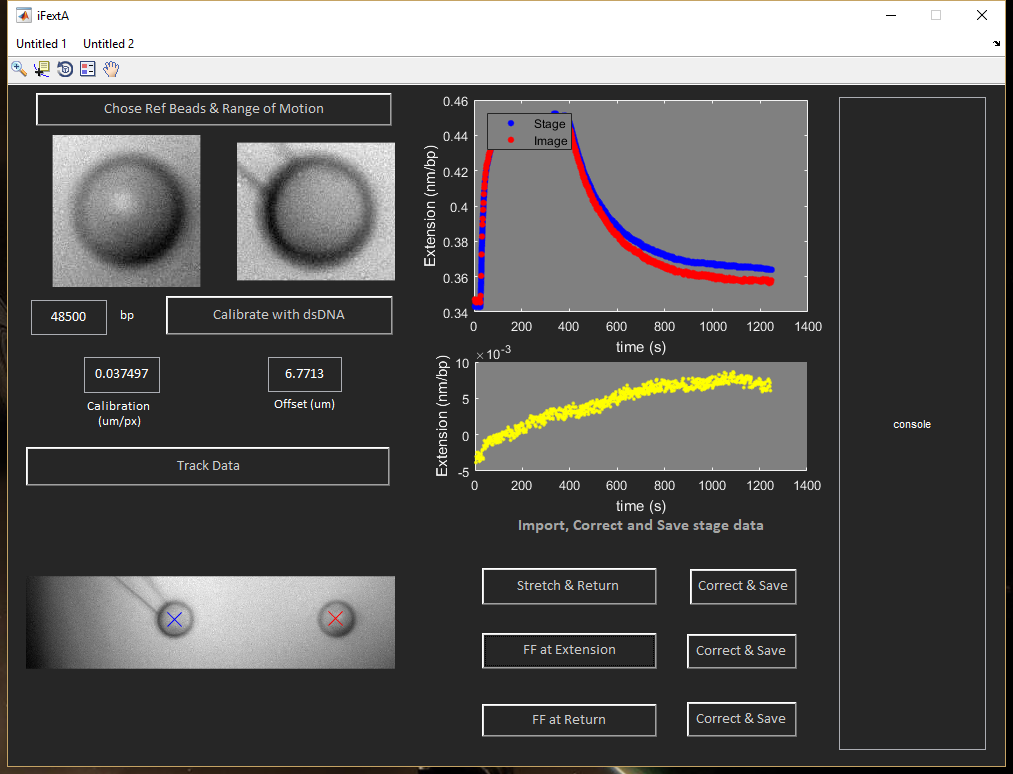

Supplement: Source code 3. [file elife-52649-code3.zip › imFexta-master/gui.png]
