# Supplementary material for: HIV restriction factor APOBEC3G binds in multiple steps and conformations to search and deaminate single-stranded DNA
Source: eLife. 2019 Dec 18;8:e52649. doi: 10.7554/eLife.52649 (PMC6946564; doi:10.7554/eLife.52649)
Supplement: Source code 2. [file elife-52649-code2.zip › Fexta2016-master/help.docx]

Meka Hadapan !!!!!!!!!
